# Effects of the COVID-19 pandemic on the emotional disorders of adolescents from rural high schools in Dobrogea, Romania

**DOI:** 10.3389/fpsyt.2025.1717336

**Published:** 2026-01-07

**Authors:** Rodica Gabriela Enache, Claudia Sălceanu, Raluca Silvia Matei, Constantina-Alina Ilie (Miloş)

**Affiliations:** Faculty of Psychology and Educational Sciences, Ovidius University, Constanta, Romania

**Keywords:** adolescents, COVID-19 pandemic, Dobrogea, emotional disorders, externalizing disorders, internalizing disorders, Romania, rural high schools

## Abstract

**Background:**

The COVID-19 pandemic disrupted adolescents’ daily routines and access to support, with rural areas facing compounded risks due to limited digital infrastructure and scarce mental health services. This study examined differences between adolescents’ self-reports and parents’ reports of emotional disorders among high school students from rural Dobrogea, Romania.

**Methods:**

A cross-sectional study carried out (February 2022–February 2024) including 300 participants: 150 adolescents (99 girls, 51 boys; 15–18 years) and one parent for each adolescent (120 mothers, 30 fathers). Parents completed the Adolescent Symptom Inventory-4 (ASI-4) and adolescents completed the Adolescent Psychopathology Scale–Short Form (APS-SF). Descriptive statistics and non-parametric Wilcoxon signed-rank tests compared responses; multiple regressions examined the extent to which parental ratings predicted adolescent self-ratings.

**Results:**

Systematic informant discrepancies emerged for both externalizing and internalizing domains. Parents reported higher levels of conduct disorder (M = 56.94, SD = 29.87 vs. M = 50.65, SD = 10.03) and oppositional defiant disorder (M = 53.21, SD = 13.43 vs. M = 44.23, SD = 10.70), whereas adolescents reported more major depressive symptoms (M = 47.74, SD = 12.16 vs. M = 42.00, SD = 3.05). Parents also rated generalized anxiety as more severe (M = 59.10, SD = 14.78 vs. M = 46.42, SD = 12.99). Wilcoxon tests indicated significant median differences for conduct disorder (z = 2.865, p = 0.004), generalized anxiety (z = −2.397, p = 0.017), and major depressive disorder (z = 9.392, p < 0.001), but not for oppositional defiant disorder (z = 1.259, p = 0.208). Regression models showed that parental ratings significantly predicted adolescent self-ratings for externalizing and internalizing symptoms (e.g., oppositional defiant disorder (ODD) model R² = .547; generalized anxiety disorder (GAD) model R² = .603; major depressive disorder (MDD) model R² = .529), with cross-domain influences suggesting interdependence between externalizing and internalizing manifestations.

**Conclusions:**

Adolescents and parents in rural Dobrogea diverge in their perceptions of emotional difficulties following the COVID-19 pandemic, with parents tending to overestimate externalizing and anxiety symptoms and adolescents endorsing more depressive symptomatology. Findings underscore the need for multi-informant assessment and context-specific, school-connected mental health services in rural settings.

## Introduction

Emotional and behavioral disorders in adolescence intensified globally after the COVID-19 pandemic (1), as prolonged isolation heightened fear of illness (2), anxiety (3), and overall psychological vulnerability (4–7). Depression during adolescence is influenced by emotional regulation (8) and self-control capacities (9), both undergoing substantial developmental changes with long-term implications for adulthood (10). Persistent negative emotional states have been associated with academic disengagement and burnout (11), findings replicated worldwide in studies describing increased rates of depression, anxiety (12), and distress linked to prolonged social isolation and uncertainty (13). Pandemic-related economic instability further strained family dynamics, with financial hardship predicting conflict, lower emotional well-being, and increased adolescent depressive symptoms, as mediated by parental stress and work–family imbalance (14–17). Adolescents from economically vulnerable families experienced chronic stress, anxiety, and emotional exhaustion due to household burdens and unstable living conditions (18).

Disruption of peer interactions - central to social learning and emotional support—contributed significantly to increased depression and loneliness among adolescents (13), with some pandemic-related emotional disorders persisting without intervention. This underscores the importance of accessible counselling, school-based mental health support, and targeted prevention programs (19). Gender-specific patterns are notable: girls show higher rates of internalizing problems such as low self-esteem, mood and sleep disturbances, and early pregnancy-related psychosocial vulnerabilities (20), whereas boys are more prone to externalizing behaviors, including aggression, delinquency, and academic failure (21). These vulnerabilities are intensified in rural settings characterized by limited resources and delays in recognizing mood disorders, which often present atypically through irritability, school refusal, or conduct symptoms (22, 23). Bipolar disorder is frequently misinterpreted as misconduct due to overlapping symptoms such as hyperactivity and impulsivity, pointing to a need for adolescent-specific diagnostic training (24). Comorbidity between internalizing disorders (e.g., major depressive disorder) and externalizing disorders (e.g., conduct disorder) is well documented (25), and symptom overlap with normative adolescent behavior complicates differentiation from typical developmental changes (26). Substance and alcohol use have also risen among rural adolescents - particularly males aged 15 - 17 - with alcohol use exceeding 30% (27), alongside increasing access to cannabis and synthetic substances even in remote communities (28). Effective responses require comprehensive programs integrating sexual and reproductive health education, substance-use prevention, and access to mental health services, as early motherhood and depression may perpetuate cycles of poverty and social exclusion (21, 29, 30).

In Romania, adolescents similarly experienced increases in emotional disorders (31), loneliness and social disconnection (32), and elevated anxiety and depressive symptoms during lockdown (33), with these manifestations closely linked to low self-esteem (32). Although overall national prevalence rates remained relatively stable between 2020 and 2021 (34), socio-economic disparities and limited access to mental health services amplified risks (35), particularly among youth with prior trauma, lower educational attainment, younger age, and specific gender profiles (36). Reports suggest that over 40% of the population experienced anxiety or depressive symptoms, disproportionately affecting young people and women, driven by uncertainty and media-related informational pressure (37, 38). Consistent with international trends (26), Romanian girls show higher internalizing vulnerability (39), while boys present elevated externalizing patterns such as aggression, truancy, oppositional behavior, academic underperformance, and substance use (39). Diagnostic challenges also persist: in bipolar-spectrum presentations, symptoms such as pressured speech, decreased need for sleep, impulsivity, and disinhibition may mimic conduct problems, risking misdiagnosis without careful assessment of periodicity and cardinal manic features (40). Aggressive conduct is associated with male gender, absenteeism, poor academic performance, and episodic heavy drinking (41), while parental emotion-regulation interventions have demonstrated effectiveness in reducing externalizing behaviors (42).

Romania’s rural adolescents confront cumulative vulnerabilities stemming from persistent socio-economic disadvantage (22, 43) with European and national monitoring showing higher rates of anxiety, depression, and deteriorating well-being among girls in disadvantaged rural settings (30, 44). Elevated rates of adolescent pregnancy, STIs, substance use, and youth crime further highlight significant gaps in education, healthcare, and opportunity (30, 45). National demographic data show persistently high rates of childbirth among girls under 18 in rural areas (34), many associated with unplanned pregnancies and increased risk for social marginalization, emotional trauma, and postpartum depression (46). Despite improvements in women’s education and declining infant mortality (47), neonatal outcomes remain poorer in rural regions due to limited healthcare access (48). Moreover, pandemic-related deficiencies in digital infrastructure exacerbated educational disparities and psychological stress among rural adolescents (49), with school closures further reducing access to social support and mental health services typically provided through educational institutions (50). In response, various NGOs and educational institutions launched online counselling programs and mental health awareness initiatives (51).

## Materials and methods

### Study design and objectives

This study employed a cross-sectional design conducted between February 2022 and February 2024 in rural high schools from Constanţa County, Romania. The primary objectives were:

O_1_. To investigate differences in social perceptions of adolescents and their parents regarding the intensity and form of manifestation of adolescents’ externalizing emotional disorders (conduct disorder (CD) and oppositional defiant disorder (ODD)).

O_2_. To examine differences in perceptions between adolescents and parents about internalizing emotional disorders (generalized anxiety disorder (GAD) and major depressive disorder (MDD)).

### Research hypotheses

H_1_. It is hypothesized that there are differences in the social perception of adolescents and their parents regarding the intensity and form of manifestation of adolescents’ externalizing emotional disorders (conduct disorders and oppositional defiant disorders).

H_2_. It is presumed that there are differences in the social perceptions of adolescents and their parents regarding the intensity and form of manifestation of adolescent internalizing affective disorders: generalized anxiety disorder and major depressive disorder.

### Participants and setting

The study sample consisted of 300 participants: 150 adolescents (99 girls and 51 boys) aged between 15 and 18 years (the oldest participant was 18 years and 4 months old), and one parent for each adolescent (120 mothers, aged 32–52, and 30 fathers, aged 33–58). The sampling frame comprised students enrolled in grades 9–12 in public high schools located in rural localities of Constanţa County, Romania, whose parents had a low educational level (0–10 years of schooling). All rural high schools that met the eligibility criteria and whose principals and school counsellors provided institutional approval were included in the study.

Within each participating school, all adolescents in the selected classes who met the eligibility criteria were invited to participate together with one parent or primary caregiver. Inclusion criteria were: (a) adolescents aged 15–18 years; (b) enrolment in a rural high school in Constanţa County; (c) residence in a rural locality; and (d) at least one parent whose highest completed level of education was between 0 and 10 years of schooling. Exclusion criteria were: (a) adolescents whose parents had more than 10 years of schooling; (b) adolescents enrolled in urban schools or residing in urban areas; and (c) adolescents attending schools outside Constanţa County.

Participants were recruited using a non-probabilistic convenience sampling strategy based on institutional availability. After obtaining approval from school administrators and school counsellors, adolescents and their parents were contacted individually and informed about the study aims, procedures, and confidentiality safeguards. Participation was voluntary and written informed consent was obtained from both parties prior to data collection. Of all eligible families approached, 75% of adolescents and 69% of parents returned complete questionnaires, representing the study’s response rate. All dyads meeting the inclusion criteria were retained in the final analysis.

The study was planned to include approximately 214 adolescent–parent dyads. According to (52), a commonly used guideline is N ≥ 50 + 8m, where *m* represents the number of predictors included in the regression model. In the present study, the regression analyses included approximately five predictors (four parental symptom ratings - Conduct Disorder, Oppositional Defiant Disorder, Generalized Anxiety Disorder, and Major Depressive Disorder - together with adolescent gender as a covariate), resulting in the requirement N ≥ 50 + 8×5 = 90 participants. The final sample of 150 dyads (300 participants) therefore exceeded these recommended thresholds, ensuring adequate statistical power for both the Wilcoxon signed-rank tests and the multiple regression analyses conducted in this study.

The data collection process took place between February 2022 and February 2024. After obtaining institutional approval from school principals and school counsellors, information sheets and consent forms were distributed to eligible adolescents and their parents either during school hours or through the schools’ secure communication channels. Questionnaires were completed in group sessions organized in the schools where public health conditions permitted, or individually through secure online links and communication platforms such as WhatsApp and Google Meet when face-to-face administration was not possible. Several participants also agreed to brief follow-up discussions to clarify items or procedures. All responses were anonymized, and only adolescent-parent dyads that met the inclusion criteria and provided complete data-corresponding to the 69% parent response rate and 75% adolescent response rate-were included in the final dataset.

### Instruments

Parents’ social perceptions of adolescent emotional disturbance were assessed using the Adolescent Symptom Assessment Questionnaire-4 (ASI-4) and the manifestation of adolescent emotional disturbance was assessed with the Adolescent Clinical Psychiatric Disorders Rating Scale - Short Form (53, 54).

The Adolescent Symptom Assessment Questionnaire-4 (ASI-4) is a screening instrument that assesses the most prevalent psychiatric disorders manifested in adolescents aged 12–18 years. The items included in the ASI-4 are based on the diagnostic criteria set forth by the (53) in the Diagnostic and Statistical Manual of Mental Disorders (DSM).

The ASI-4 enables the assessor to gather information about the adolescent from various contexts, with both a parent version and a teacher version. The ASI-4 is a screening rather than a diagnostic tool, it assesses risk for the following disorders: ADHD the three types; Conduct Disorder; Antisocial Personality Disorder; Oppositional Defiant Disorder; Generalized Anxiety; Specific Phobia; Panic Attacks; Obsessions, Compulsions; Post Traumatic Stress; Motor and Vocal Tics; Somatization; Social phobia; Separation anxiety; Schizoid personality; Schizophrenia; Nocturnal enuresis; Enuresis, encopresis; Major depressive disorder; Dysthymic disorder; Bipolar disorder; Anorexia nervosa; Bulimia nervosa, Substance use (53, 54).

Adolescents’ social perceptions of the emotional disorders of the presenters were assessed with the Adolescent Clinical Psychiatric Disorders Rating Scale-Short Form (54), which is an instrument for assessing psychopathology and psychosocial problems experienced by adolescents aged 12–18 years. The 115 items of the APS-SF directly assess symptoms specific to the clinical disorders covered in the Diagnostic and Statistical Manual of Mental Disorders, Fourth Edition (53), as well as other problems and behaviors that interfere with psychosocial adjustment and personal competence.

Parental perceptions of adolescents’ emotional and behavioral symptoms were assessed using the *Adolescent Symptom Inventory-4 (ASI-4)*, Romanian validated adaptation (55). The ASI-4 is a screening instrument designed to evaluate the frequency and severity of symptoms associated with the most prevalent psychiatric disorders of adolescence, based on DSM diagnostic criteria. The Romanian version was adapted and standardized through a rigorous multi-step process that included linguistic equivalence, psychometric validation, and the development of national norms for both boys and girls, coordinated by David, Miclea, Albu, and Bălaj and published in the official Romanian manual. The parent-report form used in this study provides dimensional assessments of externalizing and internalizing symptoms without making diagnostic determinations, in line with the instrument’s purpose to identify clinically relevant risk levels.

Adolescents’ self-reported symptoms were assessed using the *Adolescent Psychopathology Scale – Short Form (APS-SF)*, Romanian validated adaptation (54). The Romanian edition, coordinated and adapted by Bălaj and Albu and published by ASCR, offers standardized procedures, national norms, and psychometric indices supporting its use in both research and applied settings. The APS-SF measures a broad spectrum of psychopathological manifestations relevant to adolescence, including affective, anxiety, behavioral, and adjustment-related symptoms. In this study, the subscales corresponding to the constructs under investigation demonstrated acceptable to excellent internal consistency, as indicated by Cronbach’s α coefficients computed for the present sample.

Both instruments were administered in accordance with the procedures described in their Romanian adaptation manuals. Their combined use enabled a multi-informant assessment structure that allowed for systematic comparison between parental reports and adolescents’ self-perceptions, thereby supporting the study’s objectives concerning cross-informant discrepancies.

Internal consistency was examined for all subscales included in the present analysis. In this sample, reliability coefficients were acceptable to excellent, with Cronbach’s α = 0.70 for Conduct Disorder, α = 0.86 for Oppositional Defiant Disorder, α = 0.85 for Generalized Anxiety Disorder, and α = 0.90 for Major Depressive Disorder, indicating adequate psychometric properties. The Romanian versions of both instruments were used according to their validated adaptation manuals.

### Operational definitions

Clinical constructs were operationalized as continuous dimensional scores derived from the corresponding ASI-4 (parent report) and APS-SF (adolescent report) subscales. Conduct disorder (CD) scores reflected the frequency and severity of aggressive, rule-breaking, and antisocial behaviors. Oppositional defiant disorder (ODD) scores indexed irritability, argumentative behavior, and defiance toward authority figures. Generalized anxiety disorder (GAD) scores captured chronic worry, tension, and difficulties controlling anxious thoughts. Major depressive disorder (MDD) scores assessed dysphoric mood, anhedonia, cognitive distortions and somatic symptoms. Higher scores indicated greater symptom severity, without applying diagnostic thresholds, consistent with recommended dimensional approaches for adolescent psychopathology.

### Procedure

The data collection process for this study started in February 2022 and concluded in February 2024. Participants were selected from various high schools in rural areas in Constanţa, Romania. To set up contact with the participants, the researcher utilized communication platforms such as WhatsApp and Google Meet. Several participants accepted the invitation to take part in interviews.

### Data preparation

Raw subscale scores were screened for missing values (<5%), which were handled using person-level mean imputation restricted to cases with at least 80% item completion per subscale. Assumptions for inferential analyses were examined through Shapiro–Wilk and Kolmogorov–Smirnov tests, supplemented by Q–Q plot inspection. Several variables displayed significant deviations from normality, including positive skewness in parent-reported externalizing symptoms.

### Analytic strategy

To evaluate parent–adolescent discrepancies, difference scores were calculated for each clinical construct using the formula Adolescent score – Parent score. Positive values indicated higher adolescent self-reported symptoms, whereas negative values reflected higher parental ratings. Given the paired structure of the data and the non-normal distribution of difference scores, the Wilcoxon signed-rank test was employed as an appropriate non-parametric alternative for within-dyad comparisons (56).

To examine whether parental symptom ratings predicted adolescents’ self-reported symptoms, multiple linear regression models were estimated separately for the externalizing (CD, ODD) and internalizing (GAD, MDD) domains. Predictor variables included the parent-reported subscale scores corresponding to each clinical domain, with adolescent gender entered as a covariate. All predictors were entered simultaneously (enter method) to reflect the conceptual overlap among symptom clusters. Model assumptions were assessed through evaluation of linearity, homoscedasticity, independence of errors, and normality of residuals. Multicollinearity indicators were within acceptable limits (VIF < 2.5), supporting the stability of parameter estimates.

### Data analysis

All statistical analyses were conducted using JASP, Version 0.19.3 (57). Prior to the inferential analyses, internal consistency coefficients were computed for the ASI-4 scales employed in the present study. The resulting Cronbach’s α values indicated acceptable to excellent internal reliability, with α = .70 for Conduct Disorder, α = .86 for Oppositional Defiant Disorder, α = .85 for Generalized Anxiety Disorder, and α = .90 for Major Depressive Disorder. These coefficients meet established psychometric standards for research in clinical and developmental psychology (58).

The normality of the distributions was examined using the Shapiro–Wilk and Kolmogorov–Smirnov tests; however, given the well-documented sensitivity of these tests in larger samples (59), visual inspection of Q–Q plots was additionally performed. Distributional characteristics were further assessed through skewness values. Several parent-report variables exhibited substantial positive skewness, including Conduct Disorder (skewness = 2.739), Generalized Anxiety Disorder (skewness = 1.397) and Major Depressive Disorder (skewness = 1.563) confirming deviations from normality and supporting the use of nonparametric paired-samples procedures. Consequently, the hypotheses were tested using Wilcoxon signed-rank tests, an approach suitable for within-subject differences under non-normal conditions (56).

For H_1_, paired comparisons were conducted between adolescents’ and parents’ ratings of the externalizing dimensions (Conduct Disorder and Oppositional Defiant Disorder). For H_2_, paired comparisons were performed on the internalizing dimensions (Generalized Anxiety Disorder and Major Depressive Disorder). All tests were two-tailed, with sig. estimated at 0.05.

In response to methodological considerations regarding model robustness, exploratory regression diagnostics were also conducted. To address potential overfitting and assess redundancy among predictors, multicollinearity was examined using Variance Inflation Factors (VIFs) computed through the *Collinearity Diagnostics* option in JASP. All VIF values were below 2.5, indicating an absence of problematic multicollinearity (60). Linearity was evaluated through partial regression plots, independence of errors through the Durbin-Watson statistic, and homoscedasticity via standardized residuals plotted against predicted values. No assumption violations were identified that would compromise the interpretability of the regression models. Descriptive statistics (means and standard deviations) were calculated for all clinical variables included in the analyses.

### Ethical considerations

All procedures were conducted in accordance with the Declaration of Helsinki. Participation was voluntary, and informed consent was obtained from both parents and adolescents. Data was anonymized and handled confidentially. The Institutional Review Board granted ethical approval.

## Results

The research results showed that there are differences in the social perception of adolescents and their parents regarding the intensity and the form of manifestation of emotional externalizing disorders of adolescents (conduct disorder and oppositional defiant disorder), as well as in the perception about the emotional internalizing disorders of adolescents, generalized anxiety disorder, and major depressive disorder.

Parents perceive higher levels of the frequency of manifestation of oppositional defiant disorder, and adolescents perceive themselves as more depressed than their parents see; they score higher for major depression, a discrepancy consistently shown in recent studies, indicating that parents tend to observe more externalizing behaviors, while adolescents report higher levels of internalizing symptoms such as depression (61, 62).

Descriptive statistics, as shown in **Table 1**, reveal some discrepancies between parent and adolescent ratings of externalizing and internalizing symptoms associated with externalizing and internalizing disorders. Parents reported higher conduct disorder symptoms (M = 56.94, SD = 29.87) than did the adolescents themselves (M = 50.65, SD = 10.03). Similarly, oppositional defiant and oppositional defiant behaviors were rated more by parents (M = 53.21, SD = 13.43) than by adolescents (M = 44.23, SD = 10.70). In the domain of internalizing disorders, generalized anxiety was perceived as more intense by parents (M = 59.10, SD = 14.78) compared to adolescent ratings (M = 46.42, SD = 12.99). In contrast, adolescents reported higher levels of major depressive symptoms (M = 47.74, SD = 12.16) than those attributed by parents (M = 42.00, SD = 3.05).

**Table 1:** Descriptive statistics results for parent and adolescent reports.

Scale	Parent M (SD)	Teen M (SD)
Conduct Disorder	56.94 (29.87)	50.65 (10.03)
Oppositional Defiant Disorder	53.21 (13.43)	44.23 (10.70)
Generalized Anxiety Disorder	59.10 (14.78)	46.42 (12.99)
Major Depressive Disorder	42.00 (3.05)	47.74 (12.16)

M, mean; SD, standard deviation.

Hypothesis 1 presumed that there were differences in the social perceptions of adolescents and their parents about the intensity and form of externalizing emotional disturbances, particularly conduct disorder and oppositional-defiant disorder.

To test this hypothesis, differential scores (adolescent score minus parent score) were calculated for each construct. Normality tests (Shapiro-Wilk and Kolmogorov-Smirnov) revealed significant deviations from normal distribution for both variables, so non-parametric Wilcoxon signed-rank tests were employed.

As shown in **Table 2**, the Wilcoxon test for Conduct Disorder revealed a significant median difference between parent and adolescent ratings (T^+^ = 5157.50, z = 2.865, p = 0.004), supporting Hypothesis 1 for conduct symptoms.

**Table 2:** Normality and Wilcoxon signed rank tests for externalizing disorders.

Disorder	Kolmogorov–Smirnov D	p	Shapiro–Wilk W	p	T^+^	z	p
Conduct Disorder	0.379	< 0.001	0.489	< 0.001	5157.50	2.865	0.004
Oppositional Defiant Disorder	0.121	< 0.001	0.939	< 0.001	3911.50	1.259	0.208

T^+^, sum of positive ranks in the Wilcoxon signed rank test.

In contrast, the analysis for Oppositional-Defiant Disorder did not reveal a statistically significant difference (T^+^ = 3911.50, z = 1.259, p = 0.208), indicating that adolescents and their parents hold similar perceptions.

Multiple regression analysis (**Tables 3**, **4**) partially confirms the H1 hypothesis for externalizing disorders.

**Table 3:** Regression results for the H1 hypothesis – externalizing disorders (ODD adolescents’ opinion).

Dependent variable	Predictor (parent evaluation)	β (standardized)	t	p
Oppositional Defiant Disorder (ODD)	Conduct Disorder	–0.084	– 1.031	0.304
	Oppositional Defiant	0.627	6.148	< 0.001
	Generalized Anxiety	0.123	1.489	0.139
	Major Depression	0.146	2.125	0.035
	Gender	0.006	0.082	0.935

Model summary: R² = 0.547, Adjusted R² = 0.531, F(5,144) = 34.759, p < 0.001.

**Table 4:** Regression results for the H1 hypothesis – externalizing disorders (CD adolescents’ opinion).

Dependent variable	Predictor (parent evaluation)	β (standardized)	t	p
Conduct Disorder (CD)	Conduct Disorder	0.414	3.886	< 0.001
	Oppositional Defiant	–0.161	–1.201	0.232
	Generalized Anxiety	–0.078	–0.671	0.504
	Major Depression	0.227	2.516	0.013
	Gender	0.224	3.427	< 0.001

Model summary: R² = 0.217, Adjusted R² = 0.189, F(5,144) = 7.960, p < 0.001.

In the case of Oppositional Defiant Disorder (ODD), the regression model exhibits high explanatory power (R² = 0.547, p < 0.001), indicating that parents’ perceptions significantly account for the variation in adolescents’ self-reports. The coefficient of determination (R² = 0.547) indicates that parents’ perceptions explain 54.7% of the variance in adolescents’ self-reports. However, because this value tends to increase with each additional predictor regardless of its real contribution, the adjusted R² is used to provide a more accurate estimate. In this case, the adjusted R² = 0.532, which means that after accounting for the number of predictors, the explained variance is slightly reduced to 53.2%. This correction mechanism prevents overestimation of explanatory power by penalizing unnecessary predictors, ensuring that the model’s robustness is not artificially inflated (52, 63).

The strongest predictor is the provocative opposition reported by parents (β = 0.627, p < 0.001), which directly correlates with adolescents’ perception of their own oppositional behaviors. Also, parents’ perception of major depression symptoms has a statistically significant contribution (β = 0.146, p = 0.035), suggesting that these internalizing dimensions are indirectly reflected in the externalizing manifestations perceived by adolescents. The rest of the variables (parental behavior disorder, generalized parental anxiety, and gender) did not reach significant thresholds, which indicates a limited influence on adolescents’ perception.

For Conduct Disorder (CD), the model has moderate predictive power (R² = 0.217, p < 0.001). Significant predictors are parental perception of conduct disorder (β = 0.414, p < 0.001), parental major depression (β = 0.227, p = 0.013), and gender (β = 0.224, p < 0.001), indicating that boys are more likely to report symptoms of problematic conduct.

The significant prediction of depression symptoms reported by parents indicates an overlap between the internalizing and externalizing dimensions, emphasizing the complexity of the psychopathological profile in adolescence.

The variables Parental Opposition and Generalized Anxiety did not prove significant. Overall, these results support the H1 hypothesis but highlight that the overlap between the internalizing and externalizing dimensions may explain the differences in perception between adolescents and parents.

Hypothesis 2 presumed differences between adolescents and parents in perceptions of the intensity and form of manifestation of internalizing affective disorders, namely Generalized Anxiety Disorder and Major Depressive Disorder.

As with the externalizing variables, the assumption of normality was tested for the differential scores, and the Shapiro-Wilk and Kolmogorov-Smirnov tests indicated significant deviations from the normal distribution. Wilcoxon signed-rank tests were therefore applied.

As shown in **Table 5**, the Wilcoxon test results indicated significant discrepancies between adolescent and parent-reported scores for internalizing symptoms. Adolescents reported significantly lower levels of generalized anxiety compared to parental ratings (T^+^ = 3177.50, z = -2.397, p = 0.017).

**Table 5:** Normality and Wilcoxon signed rank tests for internalizing disorders.

Disorder	Kolmogorov-Smirnov D	p	Shapiro-Wilk W	p	T^+^ (Sum of + ranks)	z	p
Generalized Anxiety Disorder	0.152	< 0.001	.872	< 0.001	3177.50	– 2.397	0.017
Major Depressive Disorder	0.344	< 0.001	.715	< 0.001	7319.00	9.392	<0.001

T^+^, sum of positive ranks in the Wilcoxon Signed Rank test.

In contrast, they rated depressive symptoms as significantly more severe than those rated by their parents (T^+^ = 7319.00, z = 9.392, p < 0.001). These results support Hypothesis 2, confirming apparent perceptual differences between the two information sources in the assessment of internalizing affective disorders.

The results of regression analyses (**Tables 6**, **7**) for internalizing disorders confirm the H2 hypothesis.

**Table 6:** Regression results for hypothesis H2 – internalizing disorders (GAD adolescents’ opinion).

Dependent variable	Predictor (parent evaluation)	β (standardized)	t	p
Generalized Anxiety Disorder (GAD)	Conduct Disorder	–0.156	–2.061	0.041
	Oppositional Defiant	0.357	3.736	< 0.001
	Generalized Anxiety	0.392	4.702	< 0.001
	Major Depression	0.257	4.009	< 0.001
	Gender	0.057	0.496	0.621

Model summary: R² = .603, Adjusted R² = .589, F(5,144) = 43.661, p < 0.001.

**Table 7:** Regression results for hypothesis H2 – internalizing disorders (MDD, adolescents’ opinion).

Dependent variable	Predictor (parent evaluation)	β (standardized)	t	p
Major Depressive Disorder (MDD)	Conduct Disorder	–0.047	–0.572	0.568
	Oppositional Defiant	0.238	2.287	0.024
	Generalized Anxiety	0.352	3.880	< 0.001
	Major Depression	0.311	4.453	< 0.001
	Gender	–0.092	–1.127	0.262

Model summary: R² = 0.529, Adjusted R² = 0.512, F(5,144) = 32.301, p < 0.001.

For Generalized Anxiety Disorder (GAD), the model exhibits high explanatory power (R² = 0.603, p < 0.001). Significant predictors are parental defiant opposition (β = 0.357, p < 0.001), parental generalized anxiety (β = 0.392, p < 0.001), and parental major depression (β = 0.257, p < 0.001). These results show that parents’ perceptions of internalizing symptoms overlap primarily with adolescents’ self-assessments. However, there is also an unexpected contribution of provocative opposition, suggesting an interdependence between externalizing dimensions and adolescent anxiety.

In contrast, parents’ perception of conduct disorders has an inverse and significant relationship with self-reported anxiety (β = –0.156, p = 0.041), indicating a distortion in parental interpretation of adolescents’ symptoms.

As for Major Depressive Disorder (MDD), the model has good explanatory power (R² = 0.529, p < 0.001). Significant predictors include parental defiant opposition (β = 0.238, p = 0.024), parental generalized anxiety (β = 0.352, p < 0.001), and parental major depression (β = 0.311, p < 0.001). This model indicates that adolescents who report symptoms of depression are similarly perceived by their parents, but perceptions of externalizing behaviors (opposition) further contribute to the understanding of internalizing symptoms.

Variables related to parental conduct and gender disorders showed no significant effects. The results confirm the H2 hypothesis and highlight the importance of the concordance between adolescents’ and parents’ perceptions regarding internalizing disorders, while suggesting the existence of interconnections between the internalizing and externalizing dimensions.

## Discussion

The present study provides empirical support for systematic discrepancies between adolescent self-reports and parental evaluations of emotional and behavioral symptomatology following the COVID-19 pandemic, thereby confirming both research hypotheses. Informant divergence is well documented in the literature (64), and although traditionally interpreted as measurement error, it is now recognized as a meaningful indicator of differential access to internal states and contextualized behavioral cues. The current results expand this theoretical framework by showing that, in a rural Romanian context affected by pandemic-related disruptions, informant discrepancies manifest in distinct patterns across externalizing and internalizing domains.

For externalizing symptoms, parents reported significantly higher levels of conduct disorder manifestations compared with adolescents, whereas perceptions of oppositional defiant behaviors were congruent. This asymmetry may be understood in relation to the specific phenomenology and social salience of the behaviors assessed. Conduct disorder, characterized by aggression and violations of the rights of others (65) or breaches of established norms (66) is likely to disrupt family functioning and parental authority, making such behaviors more visible and subject to heightened parental monitoring (67, 68). In contrast, oppositional defiant behaviors are more context-sensitive and may reflect situational interpersonal dynamics rather than stable patterns of rule-breaking, which could lead to greater convergence between respondents.

The literature highlights multiple developmental and environmental mechanisms associated with conduct-related problems, including harsh or inconsistent parenting practices (69), intergenerational patterns of conduct symptoms (70), parent–child relationship quality (71), socioeconomic adversity or exposure to family violence (72), low parental warmth or overprotective control (73), limited parental mental health literacy (74), parental stress (75), attachment disruptions (76) and weak family cohesion (77). These contextual factors may amplify parental sensitivity to externalizing behaviors, especially when these behaviors threaten household stability or violate parental expectations (78). Parents may respond through corrective strategies, such as withdrawal of privileges (79), which can further influence their interpretation and reporting of behavioral problems.

Developmental factors also contribute to discrepancies. Adolescents’ increasing autonomy (80) and cognitive maturation facilitate independent appraisal of their own behaviors, while decreasing daily interaction with parents (81) reduces parental exposure to certain manifestations. Pandemic-related social isolation intensified within-family contact (82), potentially amplifying parental awareness of disruptive behaviors, while simultaneously constraining adolescents’ opportunities for autonomy-supportive social experiences. Family routines associated with reduced risk behaviors, such as shared activities or consistent parental involvement (83), may have been disrupted, and multiple adverse family conditions - including single-parent households, parental criminality, domestic violence, high-conflict environments, or cohabitation with individuals experiencing mental health difficulties-are associated with greater conduct-related problems (84).

Technology-related factors are similarly relevant. Increased reliance on digital environments during the pandemic coincided with well-established associations between problematic internet use and conduct problems (85) as well as correlations with aggressiveness, depression, and anxiety (86). Digital platforms may facilitate expression of socially unacceptable impulses, functioning as a behavioral outlet, while also reinforcing patterns of avoidance or mood regulation (87). Excessive screen exposure disrupts sleep (88), affects academic functioning (89), impairs social relationships (90), and can exacerbate interpersonal conflicts (91).

Parents’ own patterns of internet use may serve as behavioral models for adolescents (92) contributing to divergent interpretations of technology-related behaviors within the family system.

Communication processes between parents and adolescents offer another explanatory pathway. Parents typically hold more favorable views of communication quality than adolescents (93), and parental overestimation of relational functioning is associated with poorer adolescent psychological outcomes (94). Adolescents may underreport externalizing behaviors due to social desirability, impression management, or self-deception (95, 96) aimed at preserving a positive self-concept (97).

Their reports are inherently subjective and influenced by recall biases and self-awareness (98). Conversely, parental evaluations may be shaped by expectations, stress, disciplinary goals, or interpretive biases.

Emotion regulation processes also play a central role. Parental responses that are inconsistent, punitive, or emotionally unregulated can undermine adolescents’ regulatory capacities and increase the likelihood of conduct-related manifestations (99).

Adolescents may interpret parental intrusiveness as boundary violation, whereas parents may interpret adolescents’ autonomy-seeking as oppositional behavior (100). Divergent conflict-resolution strategies further contribute to perceptual discrepancies: adolescents prioritize autonomy, whereas parents emphasize relational maintenance and household order (101).

Conflicts often center on responsibilities and independence (102) and although adolescents ideally endorse compromise-based solutions, their actual conflict behavior may diverge from these ideals (103, 104). Individual personality characteristics of both parents and adolescents shape these perceptions (105).

Notably, the relative perceptual convergence observed for oppositional defiant behaviors aligns with evidence suggesting that these behaviors may be less disruptive in certain familial contexts and, therefore, more uniformly recognized across respondents (106). Neuropsychological studies also indicate that adolescents with oppositional defiant symptomatology often exhibit executive functioning difficulties and comorbid anxiety or depression (107), which may contribute to shared perceptions between parents and adolescents.

Regarding internalizing symptoms, adolescents reported significantly higher levels of depressive symptoms compared with parental evaluations, whereas parents rated generalized anxiety as more severe (108). These findings are consistent with theoretical models emphasizing differential visibility of internalizing manifestations. Depressive symptoms—such as hopelessness, withdrawal, anhedonia, or emotional numbness-are often covert and lack clear behavioral indicators (67, 109). Adolescents’ direct access to internal affective states enhances the accuracy of their self-reports, whereas parents may be unaware of or misinterpret subtle changes in mood. Conversely, anxiety symptoms, including avoidance, restlessness, tension, or irritability, present more recognizable behavioral cues, which parents may interpret as evidence of distress, while adolescents may normalize or minimize these symptoms (96, 109).

Cognitive biases may further shape adolescent self-perceptions. Depressed adolescents may misinterpret parental affect, under perceive positive cues, and over identify anger, contributing to relational misunderstandings and heightened symptom reporting (110). Although not a direct cause of discrepancy, parental depression or elevated parenting stress can contribute to adolescent distress and influence behavior within the family system (111). Parenting practices are also central: autonomy-supportive parenting fosters adolescents’ volitional functioning and self-confidence, whereas intrusive or controlling behaviors undermine self-regulation, potentially increasing anxiety symptoms (112). The quality of parent-adolescent relationships is strongly associated with depressive symptoms (113), making it plausible that relational strain during the pandemic contributed to divergent perceptions.

Cultural and contextual factors play an additional explanatory role. Evidence on rural versus urban parenting styles suggests that rural environments may be associated with more authoritarian practices rooted in traditional values and socioeconomic constraints, whereas urban contexts often endorse more authoritative, autonomy-supportive strategies (114). Lower socioeconomic status is consistently linked to more authoritarian parenting (115) and such conditions disproportionately affect rural settings (116). These contextual dynamics may shape how adolescents express and report internalizing symptoms and how parents interpret them. In urban contexts, adolescents’ stronger need for independence aligns more closely with authoritative parenting, enhancing communication and symptom recognition (117).

Overall, the current findings underscore the value of a multi-informant approach in assessing adolescent emotional and behavioral functioning. Informant discrepancies should not be dismissed as error but rather conceptualized as complementary perspectives reflecting distinct access points to psychological processes (96, 108). These discrepancies highlight the multidimensional nature of adolescent psychopathology and emphasize the importance of integrating parent and adolescent reports in clinical evaluations. The study’s results have practical implications: interventions targeting externalizing behaviors may benefit from parent training, consistent disciplinary strategies, and improved communication, while internalizing difficulties require enhanced emotional articulation, psychoeducation, and strengthened parental attunement to less observable symptoms (95, 106). Understanding differences in family perceptions is crucial for designing tailored interventions within rural communities (118).

## Conclusions

This study examined discrepancies between adolescents’ and parents’ perceptions of externalizing and internalizing emotional disorders within rural Romanian communities during the post-pandemic period. The findings indicate a consistent divergence between responses: parents tended to report higher levels of externalizing manifestations, particularly conduct-related behaviors, as well as elevated anxiety symptoms, whereas adolescents reported more pronounced depressive symptomatology. These patterns align with established evidence suggesting that externalizing behaviors are more observable to caregivers, while internalizing symptoms remain accessible only to adolescents themselves.

The results underscore the importance of relying on multi-informant assessments when evaluating adolescent mental health, especially in contexts where visibility of symptoms varies across behavioral domains. The observed discrepancies also highlight the relevance of contextual factors specific to rural environments, where limited access to mental health resources may shape patterns of symptom recognition and reporting.

Overall, the study contributes to a more nuanced understanding of how emotional and behavioral difficulties are perceived within the family system and emphasizes the need for assessment practices that integrate both adolescent and parental perspectives. Given the heightened vulnerability associated with adolescence and the additional pressures imposed by the COVID-19 pandemic, such approaches are essential for improving the accuracy of identification and guiding appropriate support strategies in school and community settings.

## Limitations, research gaps, and future directions

Although the study makes important contributions in understanding the impact of the COVID-19 pandemic on the emotional disorders of adolescents in rural areas, some limitations need to be mentioned.

Firstly, the cross-sectional design does not allow the establishment of firm causal relationships between parents’ perceptions and adolescents’ self-assessments. The results describe correlations and predictive models valid for the time of data collection, but do not capture the evolutionary dynamics of emotional symptoms.

Secondly, the study relied on self-completed reporting and parental perceptions, which can introduce biases and subjectivity. Thirdly, the research was limited to high schools in a single rural area (Constanta County), which restricts the degree of generalization of the results to other rural regions in Romania and Central and Eastern Europe (30).

These limitations open and highlight the research gap. The existing literature on the emotional health of adolescents in Romania during and after the pandemic focuses predominantly on urban settings and international samples (33, 119), leaving the situation of adolescents in rural Eastern Europe insufficiently explored.

Moreover, few studies compare adolescents’ and parents’ perceptions of the same disorders simultaneously (120, 121), which makes the two-dimensional analysis performed here an original contribution. The identified gap thus refers to the need for longitudinal and comparative studies between environments (urban and rural) and across multiple reporting sources (adolescents, parents, teachers, clinicians), to better understand the mechanisms by which the pandemic has shaped perceptions of adolescent mental health. Based on this gap, several future research directions are outlined.

Firstly, longitudinal investigations are needed to capture the trajectory of emotional disorders in adolescence and how parents’ perceptions change over time. Secondly, the inclusion of samples from several geographical and socio-cultural regions would allow testing the generalizability of the results (122). Thirdly, future research should integrate mixed methods, combining quantitative assessments with qualitative interviews, to explore in depth the differences in perception between adolescents and parents.

Finally, comparative studies that also involve teachers or mental health specialists could provide a triangulated perspective, increasing the accuracy of assessments and the relevance of educational and clinical interventions.

## Data Availability

The raw data supporting the conclusions of this article will be made available by the authors, without undue reservation.
